# Supplementary material for: Identification and functional analysis of non-coding regulatory small RNA FenSr3 in Bacillus amyloliquefaciens LPB-18
Source: PeerJ. 2023 May 15;11:e15236. doi: 10.7717/peerj.15236 (PMC10194069; doi:10.7717/peerj.15236)
Supplement: Supplemental Information 4 [file peerj-11-15236-s004.zip › KO/CK-vs-T1_map/map04350.html]

KEGG PATHWAY: TGF-beta signaling pathway - Reference pathway


|  |  |
| --- | --- |
| **TGF-beta signaling pathway - Reference pathway** |  |

[
Pathway menu
| Organism menu
| Pathway entry
| Show description
| User data mapping
]

The transforming growth factor-beta (TGF-beta) family members, which include TGF-betas, activins and bone morphogenetic proteins (BMPs), are structurally related secreted cytokines found in species ranging from worms and insects to mammals. A wide spectrum of cellular functions such as proliferation, apoptosis, differentiation and migration are regulated by TGF-beta family members. TGF-beta family member binds to the Type II receptor and recruits Type I, whereby Type II receptor phosphorylates and activates Type I. The Type I receptor, in turn, phosphorylates receptor-activated Smads ( R-Smads: Smad1, Smad2, Smad3, Smad5, and Smad8). Once phosphorylated, R-Smads associate with the co-mediator Smad, Smad4, and the heteromeric complex then translocates into the nucleus. In the nucleus, Smad complexes activate specific genes through cooperative interactions with other DNA-binding and coactivator (or co-repressor) proteins.

|  |  |  |
| --- | --- | --- |
| Reference pathway | 184% 150% 122% 100% 82% 67% 55% | 图片下载 |
